# Status of higher TGF-β1 and TGF-β2 levels in the aqueous humour of patients with diabetes and cataracts

**DOI:** 10.1186/s12886-022-02317-x

**Published:** 2022-04-05

**Authors:** Chao Gao, Xiaolei Lin, Fan Fan, Xin Liu, Huijuan Wan, Ting Yuan, Xinrong Zhao, Yi Luo

**Affiliations:** 1 First Affiliated Hospital, School of Medicine, Shihezi University, Xinjiang Uygur Autonomous Region, China; 2 Eye Institute, Eye and ENT Hospital, College of Medicine, Fudan University, Shanghai, China; 3 State Key Laboratory of Medical Neurobiology, Institutes of Brain Science and Collaborative Innovation Center for Brain Science, Shanghai Medical College, Fudan University, Shanghai, China; 4 Shanghai Key Laboratory of Visual Impairment and Restoration, Science and Technology Commission of Shanghai Municipality, Shanghai, China; 5 Key Laboratory of Myopia (Fudan University), Chinese Academy of Medical Sciences, National Health Commission, Shanghai, China

**Keywords:** Diabetes and cataract, Aqueous humour, TGF-β1, TGF-β2, TGFB1, TGFB2, Luminex liquid suspension chip detection

## Abstract

**Background:**

Transforming growth factor (TGF) is a cytokine that acts on the proliferation, migration, differentiation, and apoptosis of cells and the accumulation of extracellular matrix components. Very few studies have precisely evaluated the concentration of TGF-β in the aqueous humour (AH) of diabetic and cataract (DMC) eyes due to the low expression of proteins in the AH or other reasons. The concentrations of TGF-β1, -β2, and -β3 in the AH of the DMC group were compared with those of the age-related cataract (ARC) group.

**Methods:**

We collected AH and lens epithelium samples from 33 DMC patients and 36 ARC patients. Luminex liquid suspension chip detection was applied to detect the concentration of TGF-β1, -β2, and -β3 in the AH samples. The expression of TGFB1/2/3 in lens epithelium samples was determined by quantitative real-time polymerase chain reaction (qRT-PCR).

**Results:**

The concentrations of TGF-β1 and TGF-β2 in AH samples of DMC eyes were higher than those of ARC eyes. The differences in TGF-β1 and TGF-β2 between the two groups were statistically significant (*P* value = 0.001 for TGF-β1, *P* value = 0.023 for TGF-β2). The difference of the correlation between TGF-β1 and glycosylated haemoglobin was significant (*P* value = 0.011, and Pearson correlation coefficient = 0.306). The difference of the correlation between TGF-β2 and glycosylated haemoglobin was significant (*P* value = 0.026, and Pearson correlation coefficient = 0.269). The mRNA expression levels of TGFB1 and TGFB2 were upregulated in DMC epithelium samples compared with ARC epithelium samples. The differences in TGFB1 and TGFB2 between the two groups were statistically significant (*P* value for TGFB1 = 0.041, *P* value for TGFB2 = 0.021).

**Conclusions:**

The concentrations of TGF-β1 and TGF-β2 in AH samples were significantly higher in DMC eyes than in ARC eyes. The higher the glycosylated haemoglobin was, the higher the concentrations of TGF-β1 and -β2 were. The mRNA expression of TGFB1 and TGFB2 was significantly upregulated in DMC epithelial samples compared with ARC epithelial samples, suggesting the proinflammatory status of the anterior chamber of DMC eyes.

## Introduction

Opacification of crystalline lenses, which is called cataracts, can be caused by many factors. Diabetes is a complex metabolic disorder involving small blood vessels that can cause widespread damage to tissues, including the eyes. Patients with diabetes often suffer from cataracts. Bilateral cataracts occasionally occur with rapid onset in severe juvenile diabetes and are called true diabetic cataracts. However, true diabetic cataracts are rare. Senile cataracts in people with diabetes, which are called diabetes and cataracts in our study, are more common. The potential mechanisms for the pathogenesis of diabetic cataracts are complicated and include the p38-MAPK signalling pathway [1, 2], polyol pathway [3], and changes in inflammatory cytokines [4–10]. However, the precise mechanism of diabetes and cataracts remains unclear.

TGF-β was related to many ocular diseases, including diabetic cataracts, glaucoma, and diabetic retinopathy. It was reported that the expression of TGF-β was increased after exposure to high glucose, which might be related to the development of diabetic cataracts [11]. It was also found that the aqueous humour levels of TGF-β1 and TGF-β2 were evaluated in the eyes of patients with acute primary angle closure [12], and the TGF-β2 concentration was significantly elevated in patients with concurrent open-angle glaucoma, and complicating diabetes [13–16], as well as in patients with neovascular glaucoma secondary to proliferative diabetic retinopathy [17]. TGF-β has important roles in epithelial–mesenchymal transition  

[18]. TGF-β2-mediated fibrosis of lens epithelial cells during posterior capsule opacification could be promoted by advanced glycation end product formation [19]. In addition, TGF-β2 could effectively attenuate the activation of the MAPK/ERK/JNK and PI3K/Akt/GSK3β pathways [20]. Jagged-1/Notch signalling was reported to be activated in TGF-β2-stimulated epithelial–mesenchymal transition, and blockade of Notch signalling reversed lens epithelial cell epithelial–mesenchymal transition and lens fibrosis [21]. The miR-26a-5p/ITGAV/TGF-β/Smad3 axis was also reported to be involved in cell viability, migration and epithelial–mesenchymal transition in diabetic cataracts [22]. TGF-β was additionally reported to function in promoting epithelial–mesenchymal transition of lens epithelial cells under high glucose conditions, and the c-Src/TGF-β signalling axis in the epithelial–mesenchymal transition of lens epithelial cells might be a potential novel therapeutic target for the prevention of diabetic subcapsular cataracts [23].

Although TGF-β might play important roles in the development of diabetes and cataracts, very few studies have precisely evaluated the concentration of TGF-β in the AH of DMC eyes due to the low expression of proteins in the AH or other reasons. Recently, Luminex liquid suspension chip detection was applied to detect a large number of selected biomarkers on one membrane for samples containing very low protein concentrations [24, 25].

Therefore, we used Luminex liquid suspension chip detection to detect the concentrations of TGF-β1, -β2, and -β3 in the AH of the DMC group compared with the ARC group. In addition, we adopted qRT-PCR to test the mRNA expression of the TGFB1, TGFB2 and TGFB3 genes between DMC and ARC epithelium samples.

## Methods and Materials

### Ethics statement

The use of human AH and lens epithelium samples from cataract eyes during surgery was approved by the Institutional Review Board of Eye and ENT Hospital of Fudan University. This study was performed in accordance with the tenets of the Declaration of Helsinki for research involving human subjects. Written informed consent was obtained from every enrolled participant.

### Collection of AH and human lens epithelium samples

We collected AH (50–100 µl from each patient) and human lens epithelium samples from 33 patients with diabetes (Type 2) and cataracts (mean age 69.85 ± 6.85, aged from 55–86 years old, HbA1c 7.15 ± 1.03%, free of other ocular diseases, free of diabetes-related microvascular and macrovascular complications, no intravitreal steroids used, taking oral medications to control sugar, and lenticular opacity ranging from C3-4, NO2-3, NC2-3, and P2-3 by LOCSIII) and 36 patients with age-related cataracts (mean age 70.36 ± 8.08, aged from 54–87 years old, HbAlc 5.90 ± 0.57%, free of other ocular diseases, and C3-4, NO2-3, NC2-3, and P1-2 by LOCSIII) before cataract surgery at Eye and ENT Hospital of Fudan University. After swabbing the eyelids and the surrounding skin with disinfectant, we created a corneal paracentesis and gently inserted a 26 G needle through the paracentesis to aspirate the AH (50–100 µl) before commencing cataract surgery. All AH and human lens epithelium samples were stored in a freezer at -80 ℃ until the next step.

### Luminex liquid suspension chip detection

Luminex liquid suspension chip detection (Catalogue Number: TGFBMAG-64 K-03) was performed by Wayen Biotechnology (Shanghai, China). The TGF-β1, -β2, and -β3 Magnetic Bead Kit was used in accordance with the manufacturer’s instructions. In brief, 25 µL of each AH sample was incubated in 96-well plates embedded with TGF-β1, -β2, and -β3 magnetic beads overnight at 4 ℃, and was then incubated with detection antibody for 1 h at room temperature. Then, streptavidin-PE was added to each well for 30 min, and the values were read using the Luminex 200 system (Luminex Corporation, Austin, TX, USA).

### RNA extraction of epithelium samples and qRT-PCR

Epithelium samples were divided into 3 groups due to the low amount of RNA from the lens epithelium, among which 8 to 12 epithelium samples were pooled together in the ARC group and 9 to 13 epithelium samples were pooled together in the DMC group. Total RNA from all epithelium samples was extracted using TRIzol reagent (15,596,026, Invitrogen, Carlsbad, CA, USA) and reverse transcribed with the RT reagent Kit (RR014A, Takara Bio, Inc, Japan) according to the manufacturer’s protocol. mRNA expression was detected using a SYBR Green detection kit (RR820A, Takara, Japan) on a LightCycler 480II Real-Time PCR System (Roche, Switzerland). GAPDH was detected as the internal control. RNA expression was determined by the 2^−ΔΔCT^ method.

The forward primer of TGBF1 mRNA was GAAATTGAGGGCTTTCGCCTTAG, and the reverse primer of TGBF1 mRNA was GGTAGTGAACCCGTTGATGTCCA. The forward primer of TGBF2 mRNA was AAGCCAGAGTGCCTGAACAA, and the reverse primer of TGBF2 mRNA was GCGCTGGGTTGGAGATGTTA. The forward primer of TGBF3 mRNA was TGCCAAAGAAATCCATAAATTCGAC, and the reverse primer of TGBF3 mRNA was AGGTAATTCCTTTAGGGCAGACAGC.

### Statistical analyses

All data are shown as the mean ± standard deviation. The detections of mRNA expression in each group were repeated three times, and the interval was one week between each time. Statistical significance was analysed by two-tailed Student’s *t* test or the chi-square test using IBM SPSS 25.0 (USA). The correlations of differences between TGF-β1, -β2, -β3 and fasting glucose and glycosylated haemoglobin were analysed by Pearson correlation analysis. A *P* value < 0.05 was considered statistically significant.

## Results

### Patient characteristics in the DMC and ARC groups

Sixty-nine eyes were enrolled in the two groups, among which there were 33 eyes in the DMC group and 36 eyes in the ARC group. In the DMC group, there were 9 males and 24 females and 23 right eyes and 10 left eyes. The mean age, mean fasting glucose and glycosylated haemoglobin of the DMC group were 69.85 ± 6.85 years old, 7.58 ± 1.90 mmol/L and 7.15 ± 1.03%, respectively. In the ARC group, there were 8 males and 28 females and 25 right eyes and 11 left eyes. The mean age, mean fasting glucose and glycosylated haemoglobin of ARC group were 70.36 ± 8.08 years old, 5.88 ± 0.75 mmol/L and 5.90 ± 0.57%, respectively. There were no statistically significant differences in gender, right or left eye, or age between the two groups (Table 1). The fasting glucose and glycosylated haemoglobin levels between the two groups were statistically significant (Table 1).

**Table 1:** Patient characteristics in the DMC and ARC groups, including numbers of patients, age, gender, right or left eye, fasting glucose and glycosylated haemoglobin

	DMC group	ARC group	*P* value
Number	33	36	-
Age (years)	69.85 ± 6.85	70.36 ± 8.08	0.776
Gender			
Male	9	8	0.627
Female	24	28	
Right or left eye			
Right eye	23	25	0.982
Left eye	10	11	
Fasting glucose(mmol/L)	6.31 ± 1.34	5.55 ± 0.06	< 0.001
Glycosylated haemoglobin	7.15 ± 1.03%	5.90 ± 0.57%	< 0.001

### Different concentrations of TGF-β1, TGF-β2 and TGF-β3 in AH samples of the DMC and ARC groups

**Fig. 1:**
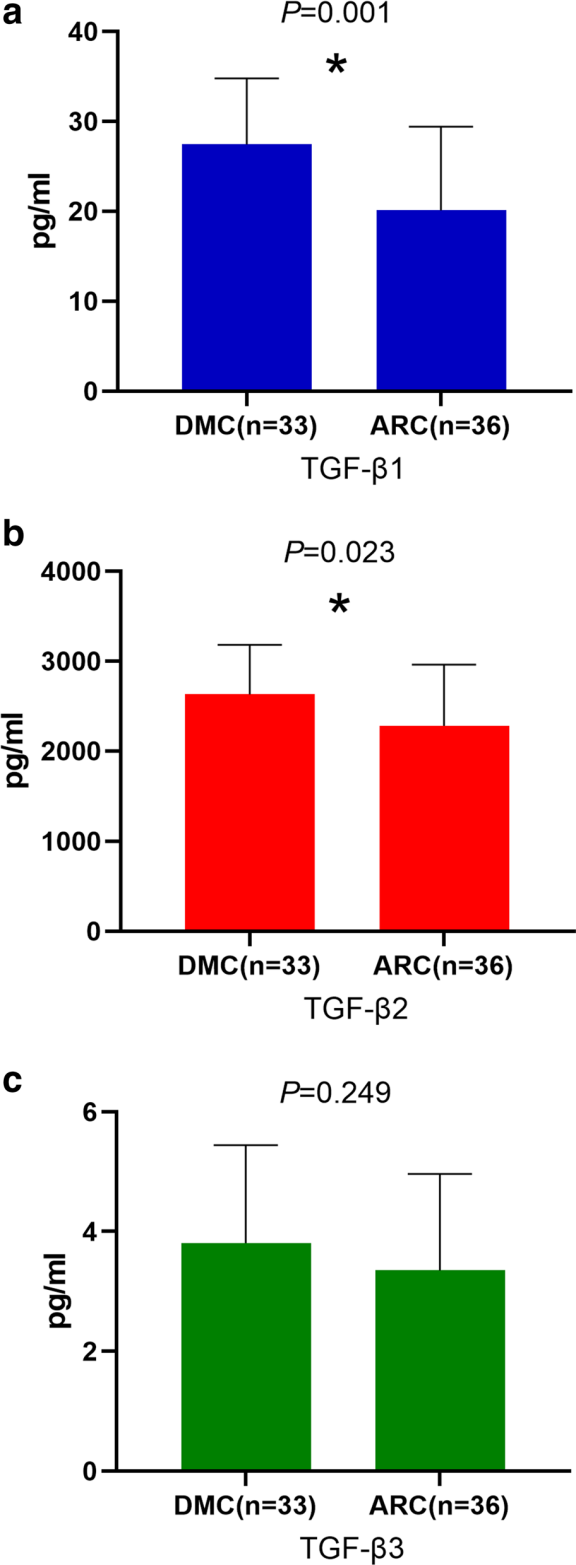
**a** Concentrations of TGF-β1 in AH samples of the DMC and ARC groups. **b** Concentrations of TGF-β2 in AH samples of the DMC and ARC groups. **c** Concentrations of TGF-β3 in AH samples of the DMC and ARC groups

We used a Luminex liquid suspension chip to detect the concentrations of TGF-β1, TGF-β2 and TGF-β3 in AH samples of the DMC and ARC groups. This detection revealed that the concentration of TGF-β1 was 27.11 ± 7.58 pg/ml in AH samples of the DMC group and 20.13 ± 9.28 pg/ml in AH samples of the ARC group; the differences between the two groups were statistically significant (*P* value = 0.001) (Fig. 1a). The concentration of TGF-β2 was 2630.55 ± 550.90 pg/ml in AH samples of the DMC group and 2281.39 ± 681.72 pg/ml in AH samples of the ARC group; the differences between the two groups were statistically significant (*P* value = 0.023) (Fig. 1b). The concentration of TGF-β3 was 3.81 ± 1.64 pg/ml in AH samples of the DMC group and 3.35 ± 1.61 pg/ml in AH samples of the ARC group; however, the differences between the two groups were not statistically significant (*P* value = 0.249) (Fig. 1c).

### The correlations of differences between TGF-β1, -β2, -β3 and fasting glucose and glycosylated haemoglobin analysed by Pearson correlation analysis

Results from Pearson correlation analysis between TGF-β1, -β2, -β3 and fasting glucose and glycosylated haemoglobin were shown in Table 2. According to Table 2, The difference of the correlation between TGF-β1 and glycosylated haemoglobin was significant (*P* value = 0.011, and Pearson correlation coefficient = 0.306). In addition, the difference of the correlation between TGF-β2 and glycosylated haemoglobin was significant (*P* value = 0.026, and Pearson correlation coefficient = 0.269).

**Table 2:** Results of correlations between TGF-β1, -β2, -β3 and fasting glucose and glycosylated haemoglobin. (*n* = 69)

N		Pearson correlation coefficient	*P* value
TGF-β1	fasting glucose	0.028	0.06
	glycosylated haemoglobin	0.306	0.011
TGF-β2	fasting glucose	0.103	0.4
	glycosylated haemoglobin	0.269	0.026
TGF-β3	fasting glucose	-0.087	0.475
	glycosylated haemoglobin	0.148	0.225

**Fig. 2:**
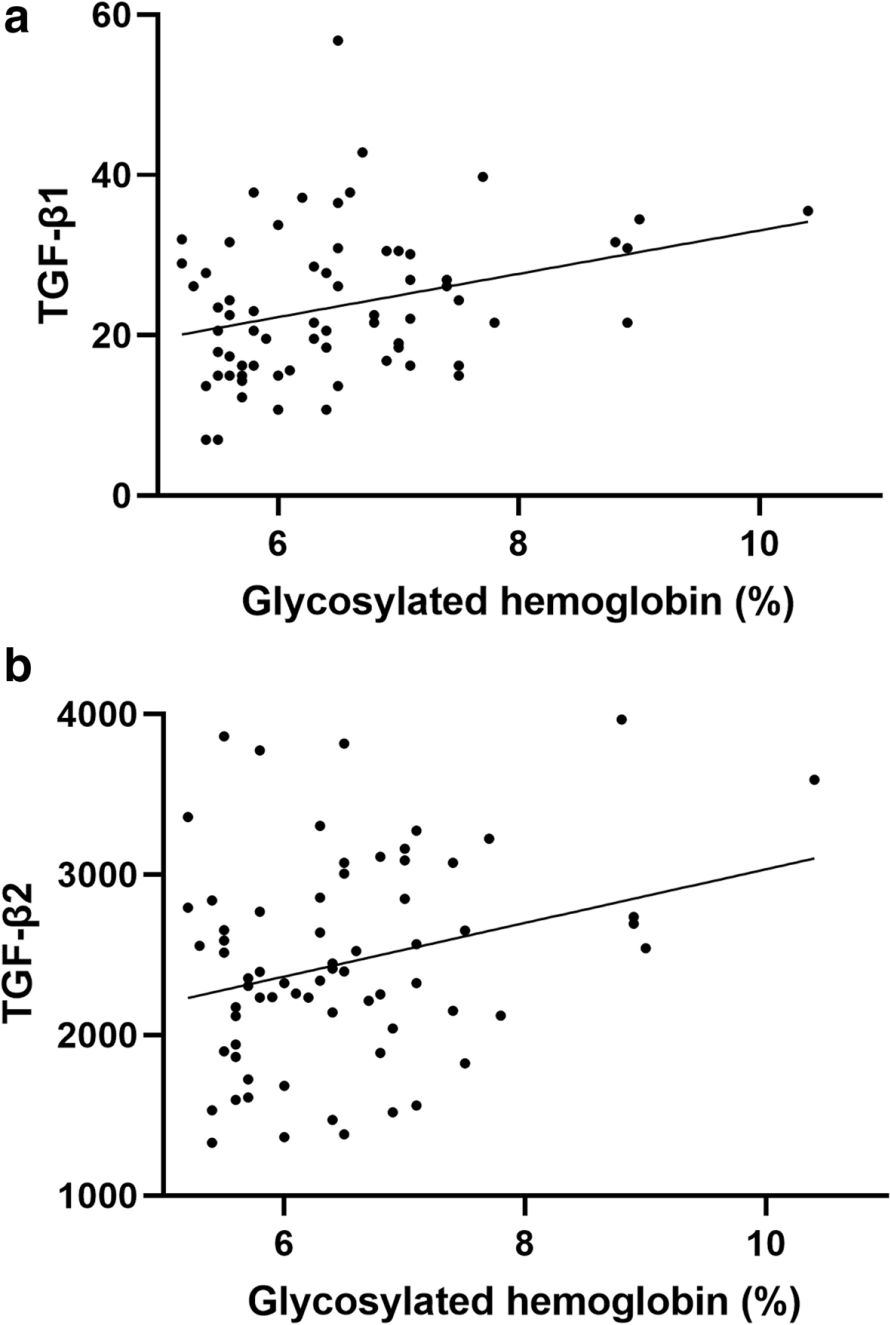
(**a**) Linear relation between TGF-β1 and glycosylated haemoglobin. (*P* value = 0.026, R^2^ = 0.094) (b) Linear relation between TGF-β2 and glycosylated haemoglobin. (*P* value = 0.011, R^2^ = 0.072)

We then made regression analysis of differences between TGF-β1 and glycosylated haemoglobin (*P* value = 0.026)(Fig. 2a), and between TGF-β2 and glycosylated haemoglobin (*P* value = 0.011)( Fig. 2b).

### Differential TGFB1 and TGFB2 expression between DMC and ARC epithelium samples

**Fig. 3:**
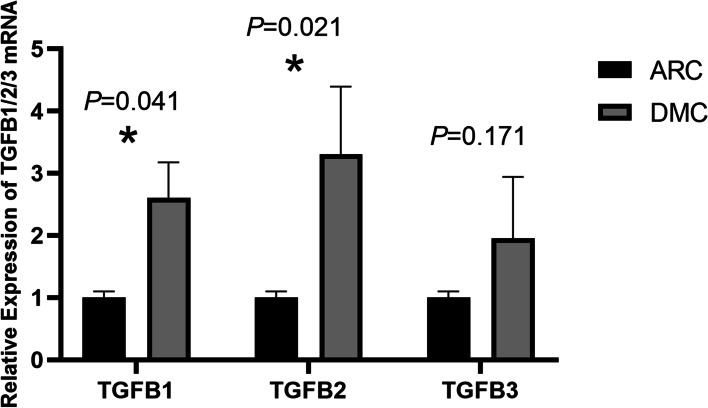
Expression of TGFB1/2/3 detected by qRT-PCR (*n* = 3 in each ARC and DMC group, and GAPDH was the internal control)

qRT-PCR was adopted to test the mRNA expression of the TGFB1, TGFB2 and TGFB3 genes between DMC and ARC epithelium samples. The expression of TGFB1 and TGFB2 was significantly upregulated in DMC epithelium samples compared with ARC epithelium samples (*P* value in TGFB1 = 0.041, *P* value in TGFB2 = 0.021) (Fig. 3). Expression of TGFB3 was upregulated in DMC epithelium samples compared with ARC epithelium samples; however, the difference between the two groups was not statistically significant (*P* value = 0.171) (Fig. 3).

## Discussion

Patients with diabetes are more likely to have cataracts. The progression of cataracts with diabetes is more rapid than that of cataracts without diabetes, although their symptoms are similar. Diabetes has long been recognized as an inflammation-related disease, and high expression of TGF-β is related to diabetes and diabetes-related ocular complications, such as diabetic retinopathy [13–17]. In the current study, we found increased expression of TGF-β1 and TGF-β2 in the AH of DMC patients compared with ARC patients by using a Luminex liquid suspension chip, suggesting a proinflammatory status in the anterior segment of DMC eyes.

TGF-β is a cytokine reported to be increased in complicating diabetes, eyes with acute primary angle closure, neovascular glaucoma secondary to proliferative diabetic retinopathy [12–17], and posterior capsule opacification [19]. AH contains numerous cytokines, including TGF-β. TGF-β was reported to be related to tissue fibrosis and epithelial–mesenchymal transition [20, 21]. Although TGF-β might play important roles in the development of diabetes and cataracts, very few studies have precisely evaluated the concentration of TGF-β in the AH of DMC eyes due to low expression of proteins in the AH or other reasons. Luminex liquid suspension chip detection could detect a large number of selected biomarkers on one membrane for samples containing very low protein concentrations [24, 25].

In our study, we used a Luminex liquid suspension chip to detect the concentrations of TGF-β1, TGF-β2 and TGF-β3 in AH samples of DMC and ARC eyes. The concentrations of TGF-β1, TGF-β2 and TGF-β3 in AH samples of DMC eyes were all higher than those of ARC eyes. However, only the differences in TGF-β1 and TGF-β2 between the two groups were statistically significant. In addition, the concentration of TGF-β2 was much higher than that of TGF-β1 and TGF-β3, which indicated that TGF-β2 might play an important role in the mechanism of diabetes and cataracts. TGF-β2 is related to fibrosis of lens epithelial cells promoted by advanced glycation end product formation [19] and is involved in several signalling pathways activated in epithelial–mesenchymal transition, such as the MAPK/ERK/JNK and PI3K/Akt/GSK3β pathways, Jagged-1/Notch signalling, and the miR-26a-5p/ITGAV/TGF-β/Smad3 axis [20–22]. Besides, TGF-β1 and -β2 were correlated with glycosylated haemoglobin, which indicated that TGF-β1 and -β2 might have important roles in diabetes and diabetes related complications. Therefore, the precise functions and mechanisms of TGF-β2 in diabetes and cataracts need to be investigated in future studies.

In addition, we tested the mRNA expression of TGFB1, TGFB2 and TGFB3 between DMC and ARC epithelium samples, which can translate TGF-β1, TGF-β2 and TGF-β3. We found that the mRNA expression levels of TGFB1, TGFB2 and TGFB3 were all upregulated in DMC epithelium samples compared with ARC epithelium samples. However, only the differences in TGFB1 and TGFB2 between the two groups were statistically significant, which was equivalent to the TGF-β1 and TGF-β2 results between the two groups. TGF-β1, -β2 and -β3 were translated by TGFB1, TGFB2 and TGFB3 genes. However, the precise reasons causing the increasing of TGF-β1 and -β2 in the AH of the DMC group remained unclear. In our future study, if possible, detection of TGF-β1, -β2 and -β3 concentrations in the vitreous humour of DMC group and a correlation analysis of TGF-β1, -β2 and -β3 concentrations between the AH and the vitreous humour might be significant.

In our previous study, we successfully isolated abundant exosomes from AH samples in the DMC and ARC groups and found that exosomal miRNAs could target the mRNAs of certain genes to affect the viability and apoptosis of human lens epithelial cells. For example, high expression of miR-551b could downregulate CRYAA expression, thus decreasing the transparency of the lens [26], and low expression of miR-29b could upregulate CACNA1C expression, resulting in an increased concentration of Ca^2+^ in the AH of DMC eyes [27]. The miR-26a-5p/ITGAV/TGF-β/Smad3 axis was also reported to be involved in cell viability, migration and epithelial–mesenchymal transition in diabetic cataracts [22]. These results indicated that increased TGF-β in the AH of DMC eyes might be activated by other factors, such as exosomal miRNAs, which might have important roles in the formation and development of diabetes and cataracts.

In conclusion, we found that the concentrations of TGF-β1 and TGF-β2 in AH samples were significantly higher in DMC eyes than in ARC eyes, and TGF-β1 and -β2 were correlated with glycosylated haemoglobin. The mRNA expression of TGFB1 and TGFB2 was significantly upregulated in DMC epithelial samples compared with ARC epithelial samples. These findings suggest that the anterior chamber of DMC eyes is characterized by a proinflammatory state that may predispose them to the occurrence of certain inflammation-related complications.

## Data Availability

All data generated or analysed during this study are included in this published article and its supplementary files.

## Abbreviations

TGF: Transforming growth factor
AH: Aqueous humour
DMC: Diabetes and cataracts
ARC: Age-related cataracts
qRT-PCR: Quantitative real-time polymerase chain reaction
